# Beyond the Acute Phase: Persistent Pulmonary Findings After COVID-19 in Hungary

**DOI:** 10.7759/cureus.90542

**Published:** 2025-08-19

**Authors:** Tania Lasrado

**Affiliations:** 1 General Medicine, East Suffolk and North Essex NHS Foundation Trust, Ipswich, GBR

**Keywords:** covid 19, covid-19 lung fibrosis, ct chest, lung pathologies, sars-cov-2

## Abstract

Objective

The objective of this one-year study, conducted at Kenézy Gyula Hospital in Hungary and involving a cohort of 50 hospitalized patients with confirmed SARS-CoV-2 infection, was to assess the long-term impact of the disease on lung morphology. Using follow-up chest CT imaging performed three months post-infection, the study aimed to identify persistent abnormalities such as ground-glass opacities (GGOs), fibrotic-like changes, and other post-inflammatory sequelae. By correlating radiological findings with clinical and laboratory data, the study sought to provide insights into the extent and nature of pulmonary recovery, highlight patterns of incomplete resolution, and contribute to the understanding of potential chronic respiratory consequences of COVID-19.

Methods

This study included 50 randomly chosen subjects. Those who suffered from COVID-19 and reported to the Emergency Department of the University of Debrecen, Clinical Center Kenézy Gyula Campus in Hungary were eligible for participation. An initial CT chest with contrast was performed on admission, and a control CT chest with contrast was performed three months following admission. The grading of affected lung parenchyma was subjective (based on the views of experienced radiologists at the department) and not AI-based or computer-guided. For comparison, the percentage of affected lung parenchyma was estimated on both the initial and control CTs. To gain a comprehensive understanding of COVID-19 infections, the subjects’ age, sex, medical history, symptoms on admission, and therapy received were also recorded.

Results

This study, based on a small sample size of 50 patients aged predominantly between 60 and 75 years, provides insights into the clinical presentation, laboratory findings, imaging features, and treatment approaches for COVID-19. The most common presenting symptoms were fever, cough, and dyspnea, with hypertension, diabetes, and cardiovascular disease being the most frequent comorbidities, factors known to influence disease severity. The most frequent findings on laboratory investigation were lymphopenia, elevated CRP, and D-dimer. This study showed GGOs with or without consolidations to be the hallmark of COVID-19 infection on CT. Regarding therapeutic methods, 74% of subjects received antibiotics (to prevent secondary bacterial infection), 44% received remdesivir, while several others received low-molecular-weight heparin to reduce the likelihood of coagulation. Supportive therapy included theophylline (bronchodilator), acetylcysteine (mucolytic), algopyrin (painkiller), and vitamin C. A great number of patients showed 30-40 (%) affected lung parenchyma on initial CT, while the control CTs depicted 5 (%) affected parenchyma.

Conclusion

Based on the results of this study, although remarkable recovery from the infection was evident, long-term effects of COVID-19 infection are apparent, with over 40% of patients exhibiting signs of distorted lung architecture. The indicators of fibrosis observed included persistent GGOs, parenchymal bands, septal thickening, and traction bronchiectasis.

## Introduction

Severe acute respiratory syndrome coronavirus 2 (SARS-CoV-2), the causative agent of COVID-19, was first identified in December 2019 in Wuhan, China [1]. Declared a global pandemic by the World Health Organization in March 2020, the virus has since demonstrated rapid human-to-human transmission, primarily via respiratory droplets and environmental contamination [1]. SARS-CoV-2 uses the ACE-2 receptor to enter host cells, which are widely expressed in the lungs and other organs, leading to systemic manifestations.

Infection typically progresses through three phases: an early viral replication phase, a pulmonary phase characterized by alveolar damage and inflammatory response, and a hyperinflammatory phase involving cytokine release and multi-organ dysfunction. Lung involvement is central to disease severity, with pathology ranging from alveolar edema and interstitial inflammation to diffuse alveolar damage and fibrosis in severe cases.

Understanding the long-term morphological changes in lung tissue following SARS-CoV-2 infection is critical, as many patients experience persistent respiratory symptoms and impaired pulmonary function, even months after recovery. This article explores the post-infectious lung morphology associated with COVID-19 and its clinical implications.

Epidemiology in Hungary

On March 4, 2020, the first two COVID-19 instances were diagnosed. The World Health Organization labeled Hungary an active center of the pandemic on March 13, 2020 [1]. The proportion of confirmed cases recorded was highest in Hungary's southwestern region, with Pécs serving as the epicenter of the infection. The elderly and those with chronic conditions were shown to have a greater infection rate, both of which are risk factors for a more severe disease course. It has also been demonstrated that the virus affects men more than women due to a higher expression of ACE-2 [2]. Symptomatic patients, particularly elderly men, were more likely to require ICU admission. These findings are supported by national-level epidemiological data from the HUN-VE Study, which reported higher ICU admissions and mortality among older adults and men with comorbidities such as hypertension, diabetes, and cardiovascular disease. The majority of asymptomatic patients recover spontaneously from a minor illness course. It was also observed that the death rate was significantly greater among the elderly, the reason being that the immune response is less efficient in older persons. In addition, as shown with SARS-CoV-1, the transition from innate to adaptive immunity is hindered in SARS-CoV-2, resulting in inadequate antibody production secondary to delayed/suppressed interferon response and dysfunction of antigen presenting cells [3].

Clinical presentation

SARS-CoV-2 infection disrupts the alveolar-capillary barrier, increasing vascular permeability and causing pulmonary edema, which impairs gas exchange, resulting in dyspnea and hypoxemia [4]. Moreover, alveolar cell injury reduces surfactant production, giving rise to alveolar collapse and hypoxia. Fever in COVID-19 is driven by cytokines (e.g., IL-1, IL-6, TNF-α) that stimulate prostaglandin synthesis [5]. Hypoxia activates peripheral and central chemoreceptors, triggering tachypnoea and tachycardia, although some patients may remain asymptomatic or have mild symptoms such as cough, dyspnea, and fever. As ACE-2 receptors are also found in the heart, blood vessels, kidneys, intestines, and the pancreas, SARS-CoV-2 can potentially invade these tissues and destroy these organs, causing multiple organ dysfunction syndrome. Other symptoms include fatigue, headache, nausea, vomiting, anorexia, chest pain, myalgia, diarrhea, and ageusia.

Non-specific screening tests

The laboratory findings most consistent with COVID-19 are lymphopenia, elevated CRP, D-dimer, and lactate dehydrogenase (LDH). Interferons are responsible for lymphopenia. Hepatocytes are stimulated by IL-6 to produce acute-phase reactants, including CRP, fibrinogen, and hepcidin. CRP is an excellent marker of inflammation. Patients with COVID-19 may have elevated D-dimer values because of acute lung injury or an elevated risk of thromboembolic complications [6]. Severe infections result in cytokine-mediated tissue destruction and the release of LDH. Elevated LDH levels are related to a greater risk of severe COVID-19 sequelae due to multiple organ injury.

Radiological findings

Chest CT scans are highly effective in detecting COVID-19, with a sensitivity of about 98%, outperforming RT-PCR tests. Common radiological findings include ground-glass opacities (GGOs), which appear as hazy areas of increased lung density without obscuring bronchial or vascular structures, typically distributed peripherally and subpleurally. Consolidations, seen as denser areas that obscure vessel margins due to fluid or cellular replacement, are also frequent, often patchy and subpleural. Reticular patterns, caused by thickened interlobular septa from lymphocyte infiltration, and crazy paving patterns - thickened septa overlaying GGOs - are other notable features. Additional findings may include traction bronchiectasis from bronchial wall damage, pleural thickening or effusions, fibrosis from healing inflammation, nodules, and specific signs such as the halo and reverse halo (atoll) signs. Overall, COVID-19 CT scans commonly show bilateral, posterior, and peripheral GGOs with or without consolidations [7].

Importantly, systematic reviews and meta-analyses have demonstrated that these radiological abnormalities often persist beyond the acute phase of infection. A meta-analysis by Fabbri et al. found that approximately 43% of patients showed residual CT abnormalities six months post-infection, most commonly GGOs and fibrotic-like changes [8]. Similarly, Wu et al. reported that up to 30% of hospitalized patients developed signs of post-COVID fibrosis, especially those who experienced severe illness or required mechanical ventilation [9]. These findings underscore the clinical relevance of long-term radiological surveillance in COVID-19 survivors.

Management 

Existing COVID-19 patient care primarily involves symptomatic treatment and supportive care including bed rest, adequate caloric and fluid intake, homeostasis maintenance, oxygen therapy, vitamin C, and probiotics [1,10]. Mild cases are managed as outpatients with instructions to recognize danger signs and prevent household transmission. The WHO recommends treating cough and fever with antipyretics, hydration, and nutritional monitoring, while cautioning against widespread antibiotic use due to risks of resistance and increased morbidity [6,10]. Hospitalized hypoxic patients receive oxygen via face masks, nasal cannulas, or noninvasive ventilation, with mechanical ventilation or extracorporeal oxygenation reserved for severe respiratory failure [10].

Anticoagulation with low-molecular-weight heparin (LMWH) is advised for patients with elevated D-dimer levels, to mitigate risks of coagulopathy-related complications [7]. Anti-inflammatory therapy including corticosteroids may reduce lung injury and respiratory failure progression but should be reserved for specific indications due to potential harm and limited evidence [1,10]. Tocilizumab, targeting IL-6 receptors, is used in severe inflammatory states such as cytokine release syndrome [10]. Antiviral agents like remdesivir inhibit viral RNA polymerase and are most effective when administered early; other antivirals such as lopinavir/ritonavir and antimalarials (chloroquine, hydroxychloroquine) have limited proven efficacy and notable adverse effects [1,10]. Type I interferons may aid early viral clearance but carry risks if administered late [4]. Respiratory support requires continuous monitoring of oxygen saturation, with escalation to invasive ventilation or prone positioning in severe cases to improve oxygenation and lung mechanics [7,10].

Post-COVID lung sequelae across populations and healthcare settings

Post-COVID pulmonary findings vary depending on patient characteristics and healthcare context, but several consistent patterns have emerged. GGOs with or without fibrosis are the most common residual radiological abnormalities, particularly in elderly patients and those with comorbidities such as diabetes and cardiovascular disease. Functional impairments, such as reduced diffusing capacity for carbon monoxide (DLCO) and restrictive spirometry patterns, have been frequently reported, especially in patients who required ICU admission or mechanical ventilation. In contrast, most individuals with mild or asymptomatic infections show full recovery without long-term respiratory sequelae. Studies have shown that outcomes are influenced by healthcare setting; high-resource environments typically offer early imaging, follow-up, and rehabilitation, which may reduce long-term damage. In lower-resource or overburdened healthcare systems, delayed interventions may contribute to more persistent lung injury. For example, Wu et al. reported that a substantial proportion of patients demonstrated residual GGOs on follow-up CT at 3 to 12 months after hospital discharge, with complete radiological resolution achieved in only a minority of cases [9]. Another systematic review and meta-analysis reported that residual radiological abnormalities, particularly ground-glass opacities and fibrotic-like changes, were common findings in post-COVID patients [11]. These findings highlight the importance of long-term respiratory monitoring, especially in older or severely affected patients.

## Materials and methods

Study design and participants

This retrospective observational study was conducted at Kenézy Gyula Hospital in Hungary over one year, from March 2020 to March 2021. A total of 50 participants were selected at random from eligible patients who presented to the hospital with signs and symptoms consistent with COVID-19. To reduce selection bias, a simple random sampling method was used to select participants from the pool of eligible hospitalized COVID-19 patients during the study period. The sampling frame consisted of all adult patients (aged 18 and over) who were admitted to Kenézy Gyula Hospital between March 2020 and March 2021 with a confirmed diagnosis of COVID-19 and who met the inclusion criteria. Patient medical record numbers were compiled into a list, and a random number generator was used to select 50 patients from this list. This approach ensured that each eligible patient had an equal probability of being included in the study. Randomization was conducted using Microsoft Excel’s RAND function.

Participants were eligible for inclusion in the study if they presented to Kenézy Gyula Hospital with clinical signs and symptoms consistent with COVID-19 infection and had a confirmed diagnosis through clinical evaluation and/or laboratory testing. Eligible patients required hospital admission for therapeutic interventions, such as supplemental oxygen therapy, and were admitted within the one-year study period. Additionally, only individuals aged 18 years or older were included.

Participants were excluded from the study if their symptoms were mild and could be managed at home or through outpatient care, or if they did not require inpatient interventions such as oxygen therapy. Individuals under the age of 18 were also excluded. Additionally, patients with incomplete medical records were excluded to ensure the reliability and consistency of the data set. Incomplete records were defined as those lacking critical clinical documentation required for the study's primary outcomes. This included missing chest CT imaging, absent or incomplete laboratory test results (e.g., CRP, D-dimer, lymphocyte counts), undocumented treatment timelines (such as antibiotic start dates or duration), or missing follow-up data such as control imaging. Only patients with a complete set of baseline and follow-up clinical, radiological, and laboratory data were included to allow for consistent and meaningful analysis of disease progression and outcomes.

Statistical analysis

Descriptive statistical methods were used to analyze the clinical, laboratory, and radiological data of the study population. Continuous variables were summarized using means and standard deviations (SD) for normally distributed data, and medians with interquartile ranges (IQR) for non-normally distributed data. Categorical variables were expressed as frequencies and percentages.

Comparisons between subgroups, such as patients with complete radiological resolution versus those with persistent lung abnormalities, were made using chi-square (χ²).

A p-value of <0.05 was considered statistically significant. Where appropriate, 95% confidence intervals (CI) were calculated to provide estimates of precision.

Ethical approval and informed consent 

This study was conducted in accordance with the ethical principles of the Declaration of Helsinki, which outlines guidelines for medical research involving human participants. The protocol underwent independent review and received approval from the Institutional Review Board of the University of Debrecen, Kenézy Gyula Campus (Ethical Approval No.: DE KK RKEB.IKEB 6336-2023).

In line with the Declaration of Helsinki's principles, measures were taken to protect the rights, safety, and privacy of participants. All patient data were anonymized prior to analysis, and no identifiable information was collected or stored. Data were accessed only by the research team and handled in compliance with institutional and national data protection regulations.

As this was a retrospective observational study using anonymized data, the requirement for individual informed consent was formally waived by the ethics committee, consistent with Article 32 of the Declaration of Helsinki, which allows such waivers when the research involves minimal risk, the rights and welfare of participants are not adversely affected, and obtaining consent would be impracticable.

## Results

This study aims to establish CT findings after SARS-CoV-2 infection that indicate the long-term impacts of the infection and how it affects an individual's quality of life after infection with the virus. The sample data was collected from 50 patients at the University of Debrecen, Clinical Center Kenézy Gyula Campus in Hungary.

The data collected was based on the following parameters: age, sex, presence of comorbidities, the duration between the onset of symptoms and the initial hospital visit, presenting symptoms, obesity, laboratory findings, initial CT findings followed by three months post-infection (control) CT, percentage of affected parenchyma observed on the initial and control CTs, whether the patient received ICU care, therapy received during infection.

Age

Based on the data collected in Table 1, the median age of participants was 62 years (IQR 57-71), with most patients arriving at the hospital aged between 60 and 75 years. This age distribution suggests that patients required hospital-level care due to symptom severity. The most commonly reported presenting symptom was shortness of breath at rest, followed by persistent chest pain or pressure.

**Table 1:** Age

Age Group	Percentage of Patients
25-55 years	22%
56-75 years	70%
>75 years	8%

Sex

As recorded in this study, more women were affected by COVID-19, with 27 women and 23 men among the participants. This contrasts with findings from larger studies conducted in China, Europe, and the United States, where men tend to show higher vulnerability and severity of disease. The difference observed in our study may be explained by the small sample size and random sampling method, which could introduce sampling variation, as well as local demographic patterns in hospital admissions during the study period. It is therefore unlikely that this female predominance reflects a true reversal of the global trend. The precise role of sex-related biological and social factors in COVID-19 vulnerability and outcomes warrants further research.

Presence of comorbidities

As depicted in Table 2, the most common comorbidity among COVID-19 patients was hypertension. This is likely due to the upregulation of ACE-2 receptors which facilitate entry of the virus into host cells. Additionally, diabetes and cardiovascular disease were also common findings due to their effect on immunity. Moreover, diabetes also increases ACE-2 receptor expression.

**Table 2:** Comorbidities COPD: Chronic obstructive pulmonary disease

Comorbidities	Percentage of Patients
Diabetes	11 (22%)
Hypertension	26 (52%)
Cardiovascular disease	11 (22%)
COPD	3 (6%)
No comorbidities	13 (26%)

First hospital visit and presenting symptoms**

As shown in Table 3, most patients arrived at the hospital within 10 days following the onset of symptoms. The most typical manifestations of the condition were fever, cough, and shortness of breath as seen in Table 4. Other notable symptoms include headache, joint pain, and fatigue.

**Table 3:** Number of Days to Hospital Presentation

Number of Days to Hospital Presentation	Percentage of Patients
Within 10 days	70%
Between two and three weeks	30%

**Table 4:** Presenting Symptoms

Presenting Symptoms	Percentage of Patients
Fever	74%
Cough	74%
Dyspnea	56%
Fatigue	28%
Headache	16%
Joint pain	16%
Diarrhea	10%
Sore throat	8%
Myalgia	6%
Chest pain	6%

Obesity**

Obesity, defined as a BMI ≥ 30 kg/m², is associated with impaired ventilation of the lung bases, resulting in decreased blood oxygen saturation, and is known to cause chronic, low-grade inflammation. This inflammatory state leads to abnormal secretion of cytokines, adipokines, and interferons, contributing to an inadequate immune response. In this study, 32% (n = 16) of participants were obese. Descriptively, a higher proportion of obese patients demonstrated persistent CT abnormalities at follow-up compared to their non-obese counterparts. While this trend is consistent with the hypothesis that obesity may exacerbate post-COVID pulmonary changes, the small sample size precludes definitive conclusions. Further research in larger cohorts is needed to clarify the relationship between obesity and long-term radiological outcomes.

Laboratory findings

The results of the majority of blood tests are typically nonspecific, but they may help pinpoint the disease’s source. As depicted in Table 5, the laboratory values reported here were measured from blood samples collected at hospital admission. The findings most consistent with COVID-19 infection were lymphopenia and elevated CRP levels. Other indicators of infection included leukocytosis and elevated LDH and D-dimer levels.

**Table 5:** Laboratory Findings

Laboratory Findings	Percentage of Patients
Elevated CRP	92%
Lymphopenia	52%
Elevated D-dimer	52%
Elevated LDH	48%
Leukocytosis	22%

Initial CT findings 

Consistent with several other studies [4,7], as seen in Table 6, this study also found GGOs, with or without consolidations, to be the hallmark of COVID-19 infection on CT. Autopsy analyses performed in other studies have shown fluid accumulation and the formation of hyaline membranes in the alveolar walls, which may be the primary pathologic cause of GGOs. Atypical findings included septal thickening (4%), parenchymal bands (6%), traction bronchiectasis (6%), and pulmonary emboli (6%).

**Table 6:** Initial CT Findings

Initial CT Findings	Percentage of Patients
Ground glass opacities	88%
Consolidation	52%
Traction bronchiectasis	6%
Pulmonary embolism	6%
Parenchymal bands	6%
Septal thickening	4%

Affected parenchyma on initial CT

The extent of affected lung parenchyma varied widely among patients. While some showed only about 5% involvement, others had as much as 30-40%, and a subset averaged 50-70% involvement. This variability is likely influenced by factors such as age and comorbidities, which can affect immune function and disease severity. In this study, the most frequent abnormalities were GGOs (hazy areas of increased attenuation without obscuring bronchial or vascular markings), consolidations (homogeneous opacities obscuring vessel margins due to alveolar filling), and fibrotic changes such as parenchymal bands and septal thickening, which indicate residual lung injury.

ICU care

The ICU admission rate in this cohort was low, with only one patient (2%) requiring intensive care. This patient was a 64-year-old man with a history of hypertension. On admission CT, he demonstrated 30-40% lung parenchyma involvement. He was admitted to the ICU for management of severe hypoxemia, required invasive mechanical ventilation, and remained in intensive care for 10 days. He was subsequently discharged from the hospital following recovery. No ICU-related mortality occurred in this study cohort.

Treatment

Supplemental oxygen is part of standard therapy for COVID-19 patients to control symptoms of hypoxia and maintain adequate oxygen saturation. As seen in Table 7, although antibiotics are not recommended for the direct treatment of COVID-19, 74% of the patients in this study received them, likely due to the effects of macrolides like azithromycin in preventing and managing secondary bacterial infections and sepsis. Antiviral therapy, particularly remdesivir, was provided to 44% of patients. At the time of writing, remdesivir was considered the most effective antiviral drug available, though cautious use was recommended in those with risk factors for high mortality and moderate-to-severe illness. LMWH was also commonly given to reduce the likelihood of coagulation, particularly in patients with elevated D-dimer levels, over 50% of patients in this cohort had elevated D-dimer upon admission. Corticosteroid therapy, particularly methylprednisolone, was provided to 18% of patients with early critical illness and/or signs of systemic inflammation. It must be noted that these treatments were often overlapping and not mutually exclusive, reflecting the need for individualized, multi-modal management. Additional medications not depicted in the graph included theophylline (bronchodilator), acetylcysteine (mucolytic), algopyrin (painkiller), and vitamin C.

**Table 7:** Therapy LMWH: Low-molecular-weight heparin

Therapy Received	Percentage of Patients
Antibiotics	74%
Antivirals	44%
Steroids	36%
Supplemental oxygen	26%
LMWH	26%

Control CT findings 

For the purpose of this study, a control CT was done for each patient three months following the resolution of the infection to note any residual or permanent damage to the lungs. As per Table 8, only 34% of patients showed complete recovery with no notable parenchymal disease. The remaining patients showed varying signs of residual parenchymal distortion: 34% showed residual GGOs, 6% showed traction bronchiectasis with residual GGOs and septal thickening, 2% showed residual GGOs and consolidations, 11% showed parenchymal bands, and 2% showed parenchymal bands with traction bronchiectasis and septal thickening. More than 40% of patients suffered from permanent lung damage of 10-70%. Of those with lasting effects of infection, 44% exhibited residual GGOs, while the rest showed other long-term signs of damage including parenchymal bands, septal thickening, and traction bronchiectasis. These are all signs of fibrosis and are usually associated with distortion of lung architecture.

**Table 8:** Control CT Findings

Control CT Findings	Percentage of Patients
Ground glass opacities	34%
Parenchymal bands	11%
Traction bronchiectasis	6%
Septal thickening	4%
Consolidation	2%

When stratified by demographic and clinical factors, persistent radiological abnormalities were more frequent among older patients, males, and those with comorbidities. In patients aged ≥60 years, 80.0% exhibited residual lung changes compared to 37.5% in those under 60 years. By sex, residual changes were observed in 69.6% of male patients and 64.3% of female patients. Patients with one or more comorbidities demonstrated a higher prevalence of residual abnormalities (70.3%) compared to those without comorbidities (57.1%). These findings suggest that age and comorbidity status, and to a lesser extent sex, may influence the likelihood of persistent post-COVID-19 lung involvement.

As shown in Table 9, chi-square testing revealed that residual CT abnormalities were significantly more common in patients aged ≥60 years (p = 0.006). No statistically significant associations were found between residual abnormalities and obesity (p = 0.416), comorbidities (p = 0.538), or sex (p = 0.837).

**Table 9:** Association Between Patient Characteristics and Residual CT Abnormalities (Chi-square)

Variable	Test Used	p-value	Significant
Age ≥ 60 years	Chi-square	0.006	Yes
Obesity	Chi-square	0.416	No
Comorbidities	Chi-square	0.538	No
Sex (Male)	Chi-square	0.837	No

**Figure 1:**
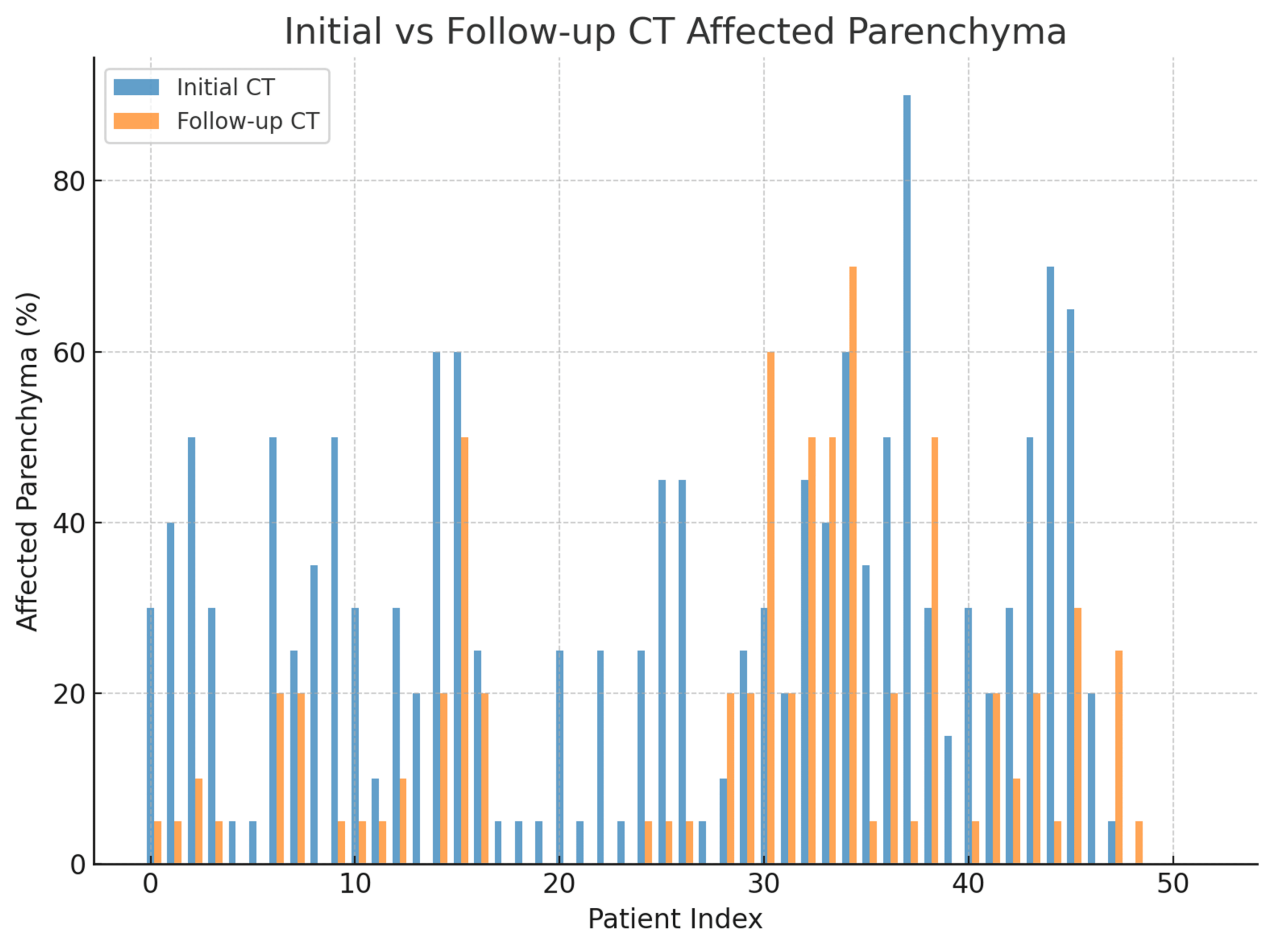
Initial Vs Follow-Up CT Affected Parenchyma

Figure 1 presents a side-by-side comparison of the percentage of lung parenchyma affected during the acute phase of COVID-19 infection (Initial CT) and three months post-recovery (follow-up CT) for each patient in the study. Overall, the figure demonstrates a marked reduction in affected parenchyma in the majority of patients, reflecting radiological improvement over time. While some patients showed complete resolution of lung changes, others exhibited persistent abnormalities, often ranging from 5 to 50% of lung involvement. A small subset demonstrated minimal change between initial and follow-up imaging, suggesting possible progression to chronic parenchymal changes such as fibrosis. This visual representation highlights both the degree of recovery and the heterogeneity of long-term lung sequelae in the cohort. 

## Discussion

People of all ages can become infected with SARS-CoV-2; however, those over the age of 60 and individuals with comorbidities such as diabetes, cardiovascular disease, hypertension, or COPD are at greater risk of developing severe illness. This increased susceptibility is most likely due to the virus utilizing ACE-2 receptors to enter host cells.

As shown by the data collected and interpreted in this study, SARS-CoV-2 infection most commonly presented with fever, cough, dyspnea, and fatigue. Obesity was included as a parameter in the study due to its role in causing low-grade inflammation, which could potentially impair immune function. Nevertheless, only 32% of the patients in the study were found to be obese.

Upon arrival at the Emergency Department of the University of Debrecen, Clinical Center Kenézy Gyula Campus in Hungary, initial CT scans and relevant blood tests were conducted for all patients. Elevated CRP emerged as the most consistent marker of infection, although it is not specific. Additionally, LDH and D-dimer levels were frequently elevated, and lymphopenia was a common finding. It should be noted that this study included only hospitalized patients, who typically present with more severe symptoms and a higher burden of comorbidities. This focus introduces potential selection bias, as the clinical and radiological findings observed here may not fully represent the broader population of individuals with COVID-19, particularly those with mild or asymptomatic disease. As such, the generalizability of these results to the community setting is limited, and future studies including non-hospitalized patients would be valuable to provide a more comprehensive epidemiological picture.

Initial CT findings suggestive of COVID-19 infection included GGOs with or without consolidations, typically involving 5-40% of lung parenchyma [7,8]. Antibiotics were commonly administered, likely to prevent secondary bacterial infections, especially considering the older age of the study population (60-75 years), which increases the risk of complications [8,12].

Control CTs showed that only 34% of patients experienced complete recovery with no residual signs of infection. Approximately 40% had persistent GGOs, a hallmark of COVID-19 infection. The results suggest that despite significant recovery, long-term effects on lung architecture are common. Over 40% of patients exhibited signs of distorted lung morphology, including persistent GGOs, parenchymal bands, septal thickening, and traction bronchiectasis. Compared with findings from larger and more diverse cohorts, the prevalence of residual changes in our study appears relatively high, which is likely related to the fact that our cohort consisted entirely of hospitalized patients, many of whom had moderate-to-severe disease and pre-existing comorbidities. In contrast, studies that also include non-hospitalized or milder cases generally report a lower frequency of persistent CT abnormalities.

Recent multicenter and meta-analytic studies have demonstrated that a significant proportion of patients recovering from COVID-19, particularly those with moderate-to-severe illness, exhibit long-term pulmonary abnormalities. A systematic review and meta-analysis by Fabbri et al. (2022) reported that over 40% of patients exhibited persistent radiological changes such as GGOs and fibrotic lesions six months post-infection, with some showing restrictive patterns on pulmonary function tests [8]. Similarly, a multinational prospective cohort study by the European Respiratory Society (ERS) found that lung diffusion capacity was reduced in over 50% of hospitalized patients at 3 to 6 months post-discharge [9].

These findings are consistent with the results observed in our study population, where residual GGOs and fibrotic features such as parenchymal bands and septal thickening were noted in over 40% of patients on follow-up CT scans. The data underscore the need for long-term respiratory follow-up and raise questions about the role of anti-fibrotic therapies in select high-risk patients.

In addition, the HUN-VE Study, a nationwide cohort analysis from Hungary, supports our findings regarding risk stratification based on age and comorbidity. It reinforces the observation that elderly males and those with underlying conditions are more prone to severe disease and, by extension, long-term pulmonary complications [12].

These structural changes may result in prolonged symptoms such as fatigue, chest pain, cough, and shortness of breath lasting for months after the initial infection. However, as this study did not include functional respiratory assessments such as spirometry, the direct correlation between radiological abnormalities and clinical respiratory impairment could not be established, limiting conclusions about the true functional impact.

However, the study had limitations. The small sample size makes it challenging to generalize the findings across a broader population, as wide confidence intervals may lead to overestimation of associations.

In conclusion, this study highlights the severe impact of COVID-19, particularly on patients with pre-existing health conditions. It underscores the need for long-term monitoring of lung health post-infection and suggests that further research is warranted to evaluate targeted interventions, such as pulmonary rehabilitation programs and the use of anti-fibrotic therapy, in severe and critical COVID-19 cases. Future prospective multicenter studies with larger sample sizes and extended follow-up periods should be undertaken to assess both the structural and functional recovery of the lungs, identify predictors of persistent damage, and evaluate the effectiveness of therapeutic strategies aimed at mitigating long-term pulmonary sequelae.

## Conclusions

The findings of this study suggest that COVID-19 may have a lasting impact on pulmonary structure, with persistent radiological abnormalities observed in a substantial proportion of patients three months after clinical recovery. Initial CT scans commonly revealed GGOs and consolidations involving a significant portion of the lung parenchyma, consistent with acute viral pneumonia. Follow-up imaging indicated that only a minority of patients achieved complete radiological resolution, while many exhibited persistent GGOs and other structural changes such as parenchymal bands, septal thickening, and traction bronchiectasis. These patterns were more frequent in older adults and those with pre-existing comorbidities, although the observational nature of this study precludes establishing causality between these findings and specific symptoms or outcomes.

This study highlights the need for long-term monitoring and proactive management of COVID-19 survivors, particularly in high-risk groups. The absence of symptom follow-up and pulmonary function testing in this cohort limits the ability to assess the functional significance of these imaging findings. Larger, multicenter, prospective studies incorporating standardized functional respiratory assessments (e.g., spirometry, diffusion capacity testing), longer follow-up periods, and more diverse patient populations are needed to better understand the trajectory of pulmonary recovery, identify patients at risk for progressive fibrosis, and evaluate the potential benefits of interventions, including pulmonary rehabilitation and anti-fibrotic therapies.
